# Digital Image Correlation Analysis of Strain Fields in Fibre-Reinforced Polymer–Matrix Composite under ±45° Off-Axis Tensile Testing

**DOI:** 10.3390/polym15132846

**Published:** 2023-06-28

**Authors:** Paweł Bogusz

**Affiliations:** Faculty of Mechanical Engineering, Military University of Technology, gen. S. Kaliskiego 2, 00-908 Warsaw, Poland; pawel.bogusz@wat.edu.pl

**Keywords:** fibre-reinforced polymer–matrix composite, vinyl-ester resin, in-plane shear test, experimental mechanics, digital image correlation method, strain map

## Abstract

This study presents an experimental investigation of an in-plane shear of a glass lamina composite using a ±45° off-axis tension test. Typically, the shear stress curve, shear modulus, and in-plane shear strength for composite lamina-type materials are identified. Previous research indicated that a loading rate affects the strength of this composite. This study extends the existing literature by utilising a non-contact optical digital image correlation (DIC) method to measure strain distribution during the test. Two cross-head displacement rates were examined. The obtained strain maps reveal an uneven distribution resembling fabric texture. As the deformation progresses, the differences in the strain pattern increase. Subsequently, a quantitative analysis of the differences between regions with extreme (minimum and maximum) strain values and regions with average values was conducted. Based on these measurements, shear stress–strain curves, indicating variations in their courses, were constructed. These differences may reach several percent and may influence the analysis of numerical simulations. The DIC results were validated using strain gauge measurements, a commonly utilised method in this test. It was demonstrated that the location of the strain gauge installation impacts the results. During the tests, the occurrence of multiple microcracks in the resin was observed, which can contribute to the nonlinearity observed in the shear stress–shear strain curve.

## 1. Introduction

In recent decades, the usage of fibre-reinforced polymer–matrix (FRP) composites as structural elements in a variety of applications due to their advantageous properties has increased. The most commonly used FRP is mainly made of glass fibre (GFRP), carbon fibre (CFRP), and aramid fibre (AFRP). In general, they offer high strength, low self-weight, ease of handling, low maintenance requirements, high durability, high tensile strength, high chemical resistance, and lower rates of aqueous corrosion. A great variety of tensile properties of various FRP materials causes some limitations during the design process. GFRP composites, in general, indicate lower tensile modulus and strength than CFRP composites; however, GFRP composites exhibit higher deformability, better impact resistance, and a much lower material cost. Therefore, GFRP composites currently dominate the reinforcement components in pultruded products due to their exceptional performance at a competitive cost [1,2,3].

Pultruded Glass Fibre-Reinforced Polymer (GFRP) refers to a composite material that is formed in the pultrusion process. In this process, continuous glass fibres are impregnated with a polymer resin, typically a thermosetting resin such as polyester or epoxy. The impregnated fibres are then pulled through a heated die, in which the resin cures and solidifies, resulting in a continuous, reinforced profile or structural shape.

GFRP is commonly used in various industries, including construction, infrastructure, aerospace, and automotive, to produce beams, columns, rods, and other structural components.

In finite element method simulations (FEM), the laminae are typically homogenised and modelled as linearly elastic-brittle orthotropic or monotropic materials. To determine the constants of elasticity, strength, and ultimate strains for FRP composites, standard tests are commonly used, or non-standard strength tests are proposed [4]. In the case of composite materials, the in-plane shear properties can be identified via a ±45° off-axis tension test. This test follows the specifications outlined in ASTM D3518/D3518M-94 and PN-EN ISO 14129 standards [5,6]. The test produces a well-known shear stress–shear strain curve with a strong non-linear behaviour. The standard procedure involves displacement-controlled loading at a crosshead displacement rate of 2 mm/min and determines parameters such as an initial in-plane shear modulus, ultimate in-plane shear strain (0.05), and in-plane shear strength corresponding to shear strains ≤0.05.

Quasi-static ±45° off-axis tension/compression tests were investigated in [7,8,9,10,11,12,13,14,15] on various FRP composite materials with thermoset or thermoplastic matrices at different crosshead displacement ratios [4]. Only short-term displacement-controlled processes were examined. The following laminae were subjected to testing: woven glass/polyester [7], unidirectional carbon/epoxy [8,9,10], unidirectional graphite/polyimide [11], unidirectional glass/epoxy [12,13], unidirectional carbon/polymer matrix [14], and stitched glass/vinyl-ester [15]. The following crosshead displacement ratios were used: 0.1 mm/min [8], 0.6 mm/min [10,14], 2 mm/min [9,12,15], and 5 mm/min [7].

In [14], the tests were conducted using a split-Hopkinson bar to investigate high strain rates. Significant increases in shear strength and yielding strength were observed along with an increase in strain rate from 5 × 10^−4^ 1/s to 1.3 × 10^3^ 1/s, while the failure strain decreased along with the increasing strain rate. This phenomenon can be attributed to the viscoelastic properties of polymers and polymer–matrix composites, as higher strength and reduced ductility were observed in epoxy compression tests conducted at higher rates. The viscoelastic properties of polymers and polymer–matrix composites were extensively discussed in numerous papers. In [16,17], the constitutive viscoelastic modelling of unidirectional glass/polyester composites was developed using a fractional exponential function. 

In [4], the authors conducted a study on a Glass Fibre-Reinforced Plastic (GFRP) composite. The off-axis tensile tests of the composite were performed at four different levels of displacement rates: 0.02 mm/min, 2 mm/min, 20 mm/min, and 200 mm/min. The objective was to demonstrate that the high nonlinearity observed in the shear stress–shear strain curve is caused by the viscoelastic flow of the resin at low shear stress levels, as well as the combined effects of viscoelastic flow and plastic microcracks in the resin at high shear stress levels. To confirm this, a classic short-term (1 h) in-plane shear creep test was conducted on ±45° off-axis samples. These samples were subjected to in-plane shear stress equal to 67% of the average in-plane shear strength obtained in the previous test.

Contemporary numerical models require precise measurements of experimental curves and strength parameters. DIC measurement is currently the most important and constantly developed non-contact strain measurement method [18,19,20,21]. Its most significant 3D version has been extended to numerous research fields, including standard materials such as metals, plastics or thermoplastics [22], advanced polymeric composites [23], and biological materials such as tissues [24] or carotid arteries [25]. Therefore, DIC has become an important method in the field of experimental mechanics [18]. 

The DIC method was used by other researchers to study the strain field of composite materials. The study conducted in [26] investigated the shear properties of carbon/epoxy composites with 0°/90° textile reinforcement structures and varying layer thicknesses under ±45° tensile loading. To analyse the deformation process on the sample surfaces during loading, an optical 2D DIC measuring method was employed. The findings indicated that shear behaviour is influenced by the layer thickness and the weight per unit area of the reinforcement. Contrary to the authors’ expectations, the optical deformation analysis of the sample surfaces revealed an uneven strain distribution.

In [27], a novel oblique end tab was designed to mitigate stress concentration and in-plane bending moments resulting from off-axis tension loading. Finite element analysis and experiments were conducted on polyetheretherketone (PEEK)/AS4 unidirectional thermoplastic composites (CFRTP) to assess the effectiveness of the proposed testing method. Simulation and test results demonstrated that the utilisation of oblique end tabs successfully eliminated stress concentration and bending movements. The digital image correlation (DIC) method was employed to investigate the deformation response, specifically tension/shear coupling, across the entire field of the off-axis specimen. The test results revealed significant non-linear behaviour and non-uniform strain distribution under combined tension/shear stresses. A fractographic examination was carried out to explore the damage mechanisms under the tension/shear combined stress state. Specimens with off-axis angles of 30°, 45°, and 60° exhibited failure in a mixed tension/shear mode.

In paper [28], DIC was utilised for tensile and bond tests on composite reinforcements with varying textiles and matrices. The results obtained from two DIC software programs were validated by comparing them with displacement and strain transducers. DIC provided additional insights into the damage pattern, such as crack location and width, as well as the load transfer mechanism between the composite and the substrate. One advantage of DIC is the ability to select multiple measurement points after the test, overcoming the limitations of traditional transducers. However, since DIC only monitors the outer surface of the specimen, direct information about the embedded textile in the matrix is not available. The combination of DIC and traditional sensors in laboratory testing allows for improving the understanding of the mechanical behaviour of composite reinforcements as well as the identification of their fundamental properties.

FRP composites are vulnerable to the presence of holes and cutouts, as several experiments have shown a significant decrease in strength. This strength degradation is primarily attributed to a stress concentration around the hole. In review [29], the objective was to understand the impact of holes on the mechanical properties of FRP laminates, with a specific focus on carbon FRP laminates and experimental findings. A comprehensive analysis of the tensile, compressive, flexural, and post-buckling properties of laminates with holes was provided to facilitate the optimal component design. The strain field near the holes obtained via DIC was compared to the output of finite element analysis, and a good agreement was observed between both sets of results.

In [30], a series of dynamic longitudinal compression tests were conducted on cross-ply IM7/8552 samples cut at different off-axis angles to produce different combinations of compression and shear stresses. Together with the results from quasi-static tests [31] performed in a similar manner, quasi-static and dynamic fibre kinking failure envelopes were obtained using a classical laminate theory. The experimental results revealed that while the leading fibre kinking theories accurately predicted the effects of a strain rate on uniaxial compression strength, they failed to account for shear effects, both in quasi-static and dynamic scenarios. The digital image correlation method was employed to validate strain gauge measurements and ensure the adequacy of the testing conditions.

FRP materials commonly employed in civil engineering exhibit significant asymmetries and heterogeneity due to manual installation procedures [32]. This study aimed to explore the applicability of an optical full-field DIC technique for strain field measurement on FRP structures in the civil engineering industry. The DIC method and more traditional measurement methods were compared. The DIC results provided valuable insights into the stress distribution around the structure. Consistent measurements demonstrated the potential of DIC in capturing rupture strains of FRP materials and understanding their capacity under the influence of these strains. Furthermore, DIC offered improved capabilities for detecting debonding effects.

The authors of [33] focused on the experimental investigation of tensile testing of CFRP-UD coupon specimens using the 10° off-axis test and DIC strain mapping to measure the full-field deformation response. A sensitivity analysis of the measured stresses, strains, and moduli was performed by varying the aspect ratio of the specimens and the location of strain measurement. Test-related uncertainties, such as the loading angle and specimen preparation details, were investigated and quantified with the use of these full-field DIC measurements.

This present study extends upon the research presented in [4]. In addition to the previous study, DIC measurements were performed on the same GFRP composite. This method enabled the measurement of strain distribution on the composite in a ±45° off-axis tension test. Similar papers can be found in the literature. However, this manuscript specifically focuses on a unique G/V composite and presents specific results. The assessment of strain maps and their comparison with the strain gauge method, which is not extensively documented in the literature, was conducted.

Two strain rates, 0.02 and 20 mm/min, were selected for this study. The measured DIC maps revealed non-uniform strain distributions. Subsequently, a quantitative analysis of the differences between regions with extreme (minimum and maximum) values and regions with average values was conducted. Based on these measurements, shear stress–strain curves, presenting variations in their courses, were constructed. These differences can reach several percent and may influence strength parameters, consequently affecting the analysis of numerical simulations. During the testing process, the formation of multiple microcracks in the resin was observed, which can contribute to the nonlinearity observed in the shear stress–shear strain curve, and, thus, supports the statement presented in [4].

## 2. Materials and Methods

**Figure 1:**
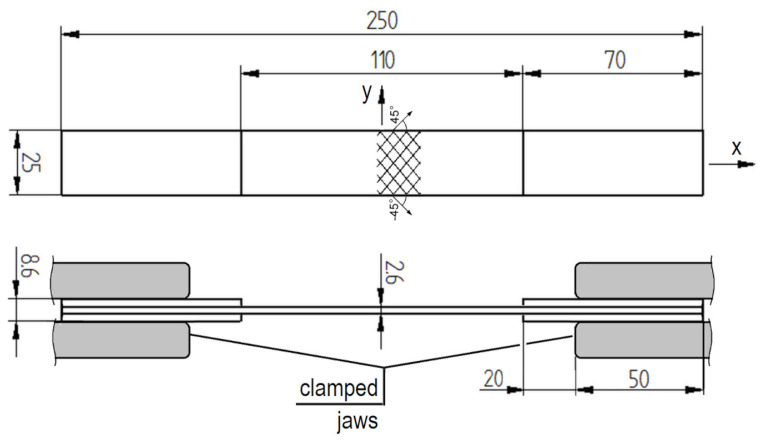
The scheme of a glass/vinyl-ester sample.

This research involved conducting ±45° off-axis tension tests to measure shear properties of flat rectangular samples of a Glass Fibre Reinforced Polymer (GFRP) composite (producer: ROMA Co., Ltd., Grabowiec, Poland) in order to analyse strain distribution. The samples were made and tested in accordance with the guidelines contained in the PN-EN ISO 14129:2000 standard [6]. The nominal dimensions of the samples were 250 × 25 mm. The overall thickness of the plate was approximately 2.6 mm. The samples had adhesive patches, with a length of 70 mm and a thickness of 3 mm, applied on both sides of the grip sections. The scheme of a sample is presented in Figure 1.

The investigated G/V composite is a layered composite made of Polimal VE-11M vinyl-ester resin (producer: CIECH Sarzyna S.A., Nowa Sarzyna, Poland), used as a matrix reinforced with GBX800 [±45] glass fabric (producer: DIPEX Co., Sereď, Slovakia). This composite design mixture is utilised for large-scale roof coverings. Glass composite is more cost-effective than carbon-reinforced composites yet still suitable for this purpose.

The G/V composite under investigation is a layered composite consisting of Polimal VE-11M vinyl-ester resin (manufacturer: CIECH Sarzyna S.A., Nowa Sarzyna, Poland) used as a matrix and reinforced with GBX800 [±45] glass fabric (manufacturer: DIPEX Co., Sereď, Slovakia). This composite is employed, among others, for extensive roof coverings. Glass composite is a more economical alternative to carbon-reinforced composites while remaining suitable for this specific application.

The Polimal VE-11M resin is a flame-retardant neutral vinyl-ester resin known for its high thermal and chemical resistance. The curing system used for 1 kg of this resin, at a temperature above 18 °C and low air humidity, includes Cobalt accelerator Co 1% (10 mL) and MEKP low reactive hardener (20 mL). Post-curing of the resin is required at an increased temperature of 80 °C for 4 h. Resin V is suitable for vacuum infusion technology [34]. The basic parameters of this resin before and after curing/post-curing are summarised in Table 1. Tensile strength is equal to 80 MPa, the tensile modulus is 3500 MPa, and relative elongation at break is 3.5%.

The GBX800 fabric (referred to as G) is a stitched E-glass fabric with a nominal surface density of 800 g/m^2^. It is quasi-balanced, orthogonal, and bi-directional fabric with a ±45 warp/weft orientation in relation to the longitudinal axis of a fabric strip and test samples.

Composite plates of G/V were manufactured by ROMA Co., Ltd., Grabowiec, Poland, using vacuum infusion technology and had a [±45]_2S_ fabric sequence. The plates were post-cured following the guidelines in [34]. The key parameters of one lamina were determined to be thickness of 0.663 mm, fibre volume fraction of 48%, and mass density of 1.70 g/cm^3^.

**Figure 2:**
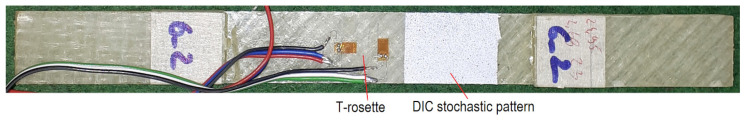
A sample with a T-rosette attached and a stochastic pattern applied.

For the measurement of shear strains, two Vishay strain gauges (producer: Vishay Precision Group, Inc., Wendell, NC, USA) with resistance of 120 Ω were installed in the central measurement zone of the samples following the T-rosette scheme, as shown in Figure 2. The strain gauges were attached along and perpendicular to the axis of the sample in accordance with standard requirements. This allowed for the evaluation of shear strains occurring in the composite fabrics oriented at a 45° angle, while the sample, itself, was subjected to axial tension.

For the purpose of DIC measurements, a stochastic black–white pattern was applied to a section of the sample used for measurements (see Figure 2). The pattern had dimensions of approximately 25 × 25 mm. The T-rosette strain gauges were located at the centre of a sample, while the DIC stochastic pattern was positioned close to this centre. It was considered essential to keep this pattern as far away as possible from the gripping area.

**Figure 3:**
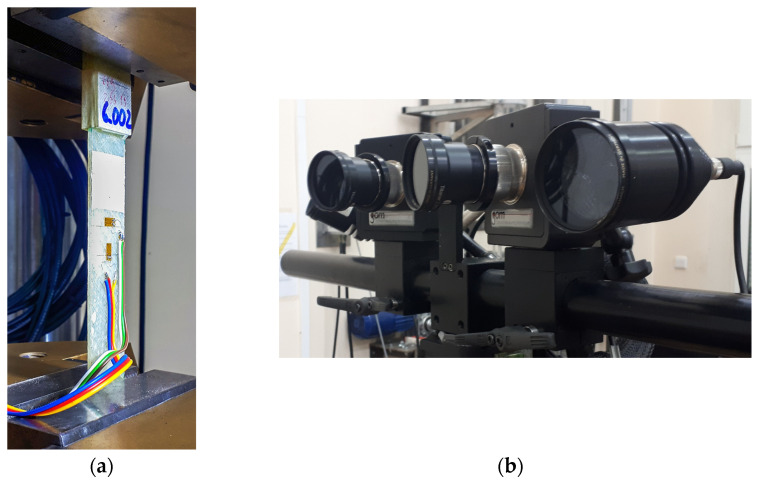
Testing setup (**a**) with DIC measurement cameras (**b**).

In the DIC measurements, the GOM Aramis system (producer: GOM mbH., Braunschweig, Germany), specifically designed for capturing deformations and strains on materials under load, was employed. The testing setup, along with cameras of the DIC system, is presented in Figure 3. The system utilises two cameras equipped with a CMOS sensor (Figure 3b) to capture a 3D image with resolution of 4 megapixels. The cameras, fitted with lenses with a fixed focal length of 50 mm, were positioned at a distance of 345 mm from the measurement object. The distance between the cameras in this configuration was 126 mm, while the cone angle was set to 25°.

The calibration procedure was applied to determine parameters such as the relative position of cameras, lens distortion, and intensity and uniformity of lighting. The system was calibrated within a measurement volume of 85 × 65 × 45 mm. The calibration deviation was 0.028 pixels.

The utilized DIC system involves the application of a virtual regular grid of regions, known as facets, onto the recorded image sequence. These facets are then correlated with the corresponding areas on the image captured using the adjacent camera at the same stage, followed by correlation with the remaining pairs of images taken. The facet size was set at 18 × 18 pixels (approximately 0.4 × 0.4 mm) with separation of 13 pixels (resulting in 2-pixel overlap). The default overlap between neighbouring facets was 2 pixels. By utilising directional strain measurements, the shear curve and related strength properties were determined. The strain maps revealed an uneven strain distribution. The DIC strain measurements and the strain gauge measurements were compared.

Shear via tension testing of rectangular GFRP composite samples was conducted at an ambient temperature of 24 °C using the Instron 8802 (producer: Instron Illinois Tool Works Inc., Norwood, MA, USA) universal strength testing machine (Figure 3a). The strains were measured using both strain gauges and the DIC method. All data was synchronised using the force signal from the testing machine. Two loading rates were tested: 0.02 mm/min and 20.0 mm/min. The frequency of the images was set at 0.05 frames/s for the slower loading rate and 5 frames/s for the higher loading rate.

Gamma strains presented in the shear curves were calculated using the procedure outlined in standard [21]:

(1) $$\gamma =\left|\varepsilon_{x}\right|+\left|\varepsilon_{y}\right|,$$

where *ε_x_* represents strain in the axial direction measured with DIC or a strain gauge method, and *ε_y_* is strain in the perpendicular direction measured with DIC or a strain gauge method.

Identical procedures were applied for both strain gauge and DIC measurements. Although the standard sets the limit for the shear strain at 5%, the tests were actually conducted until the sample fracture. Optical measurements allowed for determination of the gamma until fracture, while the strain gauges were damaged at a gamma strain level below 10%.

## 3. Results

The research presented in paper [4] aimed to investigate the influence of a loading rate on the shear stress–strain curve and material properties of the G/V composite. The determined values for material constants G_12_ and R_12_, along with their standard deviations obtained during the research, are presented in Table 2. These values were derived following the procedures described in [5,6].

It is worth noting that the shear strength is limited to a gamma strain of 0.5% on the shear curve. However, if the tests were analysed until failure, the in-plane shear strength would likely be higher. The reason for limiting the gamma strain to 5% was the assumption that the state of pure shear would be lost in such cases.

**Figure 4:**
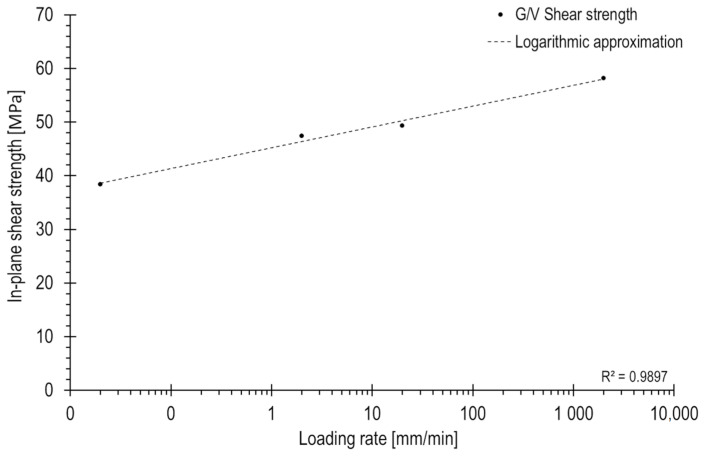
In-plane shear strength as a function of loading rate.

Based on the data presented in Table 2, a graph in Figure 4 was created to illustrate the relationship between the in-plane shear strength and the loading rate. The X-axis of the graph was scaled logarithmically to accurately represent the dependencies. The observed increase in strength is typical for polymer composites exhibiting viscoelastic properties [14]. In this representation, linear regression was applied, which resulted in a good fit to the data. The coefficient of determination for the regression is close to 1.

Directional strain maps calculated in the DIC system are presented in the subsequent figures. The maps illustrate the axial strains (direction X) corresponding to the measurements taken by the strain gauge installed parallel to the sample axis (Figure 2). In order to compare the results from both measurement methods, DIC strains in the perpendicular direction (Y direction) were also obtained. Due to the material and load symmetry, the directional strains are approximately equal to each other and are approximately half of the corresponding gamma strain, as described by Equation (1).

**Figure 5:**
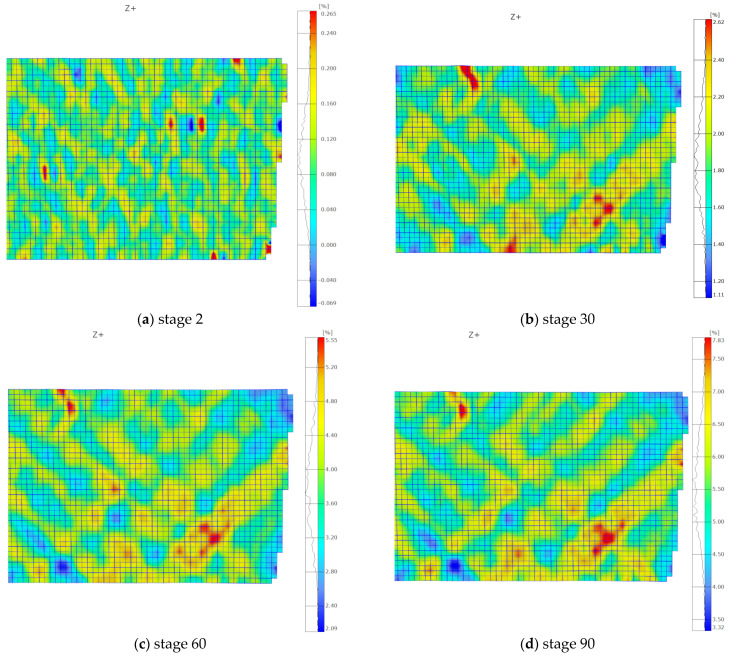
Facet maps representing strain distribution in the X direction for a sample tested at a loading rate of 0.02 mm/min: (**a**) stage 2; (**b**) stage 30; (**c**) stage 60; (**d**) stage 90.

**Figure 6:**
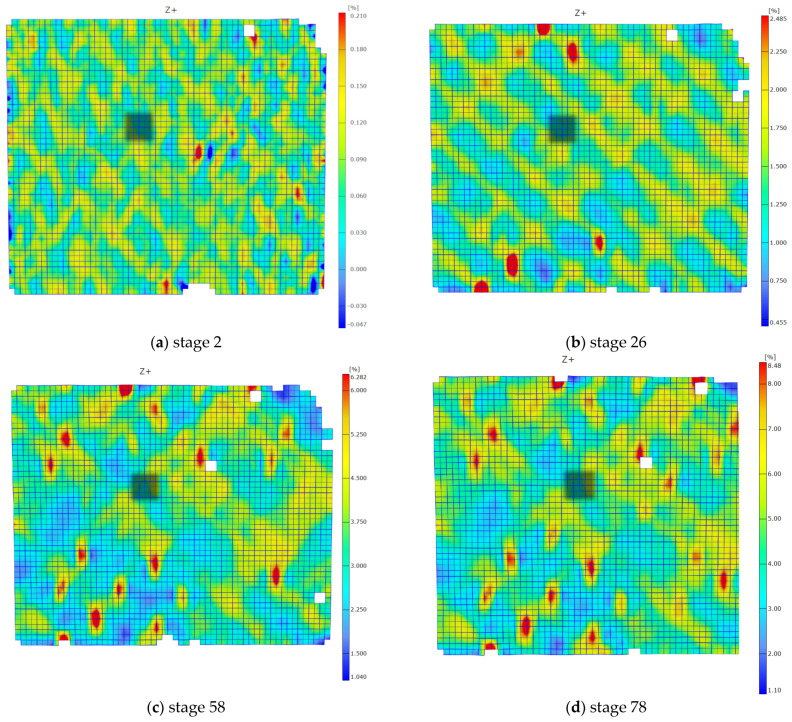
Facet maps representing strain distribution in the X direction for a sample tested at a loading rate of 20 mm/min: (**a**) stage 2; (**b**) stage 26; (**c**) stage 52; (**d**) stage 78.

The directional maps illustrating strain rates of 0.02 and 20 mm/min are presented in Figure 5 and Figure 6, respectively. Four maps representing selected stages are provided for each loading rate. The corresponding stage numbers, displayed below the maps, indicate comparable levels of shear state advancement for both loading rates. The time interval between subsequent stages depends on the set frequency. The frequency of the images on the DIC system was set at 0.05 frames/s for the slower loading rate and 5 frames/s for the higher loading rate. The time interval between subsequent stages was 20 s and 0.02 s for the 0.02 and 20 mm/min speeds, respectively.

The results reveal significant non-uniformities in the strain distribution across the measured area of the sample. The traces in the form of a net pattern, caused by the presence of the near-surface layers of glass fabric in the composite, are visible. In the first two stages, the net pattern is distinct; however, it becomes increasingly blurred in the subsequent maps. 

It is also noticeable that as the damage progresses, an increasing number of areas with higher strain values, exceeding two percent, appear in the DIC maps. These areas become more pronounced in the third and fourth images, accompanied by an increase in local strain values. During the 20 mm/min shear test, a higher number of defects were observed (Figure 6c,d) compared to the slower test (Figure 5c,d). They are distributed across the entire area and have higher strain values. This effect can be associated with the occurrence of microcracks and defects within the composite structure. In the case of the slower test, the areas seem to grow less and appear less frequently. These defects may contribute to the non-linearity of the shear curve, as described in [4]. 

**Figure 7:**
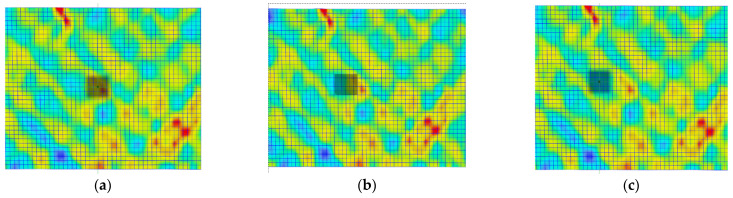
Facet fields: (**a**) area of high-value facets, (**b**) area of average-value facets, and (**c**) area of low-value facets for a sample investigated with 0.02 mm/min loading rate.

**Figure 8:**
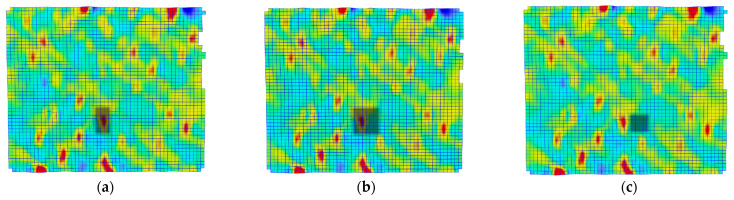
Facet fields: (**a**) area of high-value facets, (**b**) area of average-value facets, and (**c**) area of low-value facets for a sample investigated with 20 mm/min loading rate.

A quantitative analysis of these differences was conducted. Three different measurement areas of facets were selected for each tested loading rate. The areas selected are presented in Figure 7 and Figure 8, for 0.02 and 20 mm/min, respectively. The facets’ fields had dimensions of approximately 2 × 2 mm, which is comparable to the dimensions of the strain gauge used in the test (Figure 2). For each area, an average value was calculated using the DIC software. The first measurement area (labelled as “aver”) included facets with both low and high strain values, resulting in a balanced average value. The second area (labelled as “high”) was selected only from locations with higher strain facets. The third area (labelled as “low”) consisted of facets, in which lower strain values were measured. In Figure 6, the shifting of the low-value facet area during testing is illustrated.

**Figure 9:**
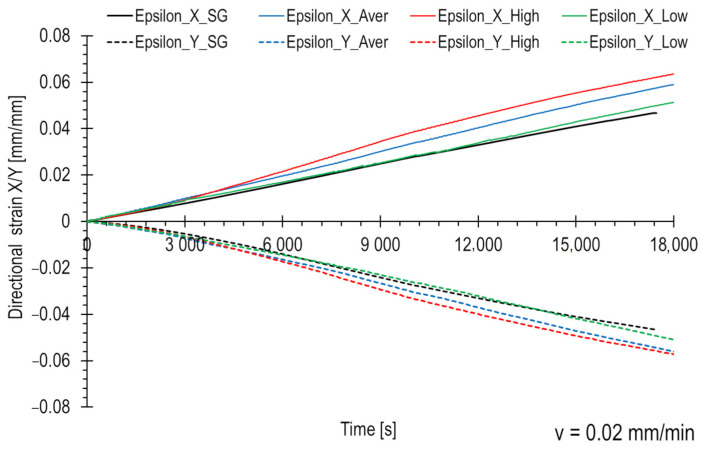
Directional strain comparison for different strain measuring methods. The loading rate is equal to 0.02 mm/min.

**Figure 10:**
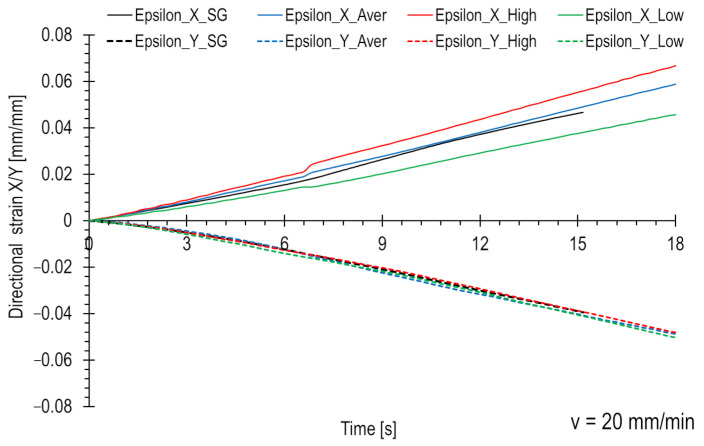
Directional strain comparison for different strain measuring methods. The loading rate is equal to 20 mm/min.

In order to quantitatively compare the strains obtained from all three groups of facets and validate them in relation to the strain gauge measurements, appropriate graphs illustrating directional strains were created. Figure 9 displays the results for a loading rate of 0.02 mm/min, while Figure 10 presents the results for a loading rate of 20 mm/min. The strain gauge measurements were presented using a black line, while the facet groups (average, high, and low) were represented by red, blue, and green curves, respectively. The XY coordinate system is assumed to lie within the plane of the composite plate, with the X-axis aligned along the sample axis and the Y-axis perpendicular to the sample axis.

The strain in the X direction is positive, while the strain in the Y direction is negative. Due to the load and symmetry material properties, the moduli of strain values are similar. A deformation range for strain gauges is limited, typically around 5%. As a result, the strain gauge curves terminate within that range. Optical methods, such as this DIC system, allow for less precise strain measurements compared to strain gauges but offer a wider strain range, exceeding even 100%. Strain gauges can accurately measure strains of a few μm/m, while the DIC system is limited to an accuracy of approximately 0.2%. However, the strain measurement range for strain gauges is narrower, about 5%. The presented curves were limited to approximately 8%. Within this range, DIC maps are also presented (Figure 5 and Figure 6). Testing at a speed of 0.02 mm/min is 1000 times longer.

As expected, higher strain values were measured in the regions with high-value facets (in terms of the modulus), corresponding to the areas depicted in Figure 7b and Figure 8b. The curves for the average-value facets (Figure 7a and Figure 8a) fall in the middle, while the curves for the low-value areas (Figure 7c and Figure 8c) are the lowest. The differences between the regions are more pronounced in the loading direction than in the transverse direction. These dependencies were observed for both tested loading rates.

The strain gauge curves in both figures lie between the high and low DIC measurements. However, it can be observed that for a velocity of 0.02 mm/min (Figure 9), the strain gauge curve closely aligns with the strains from the low-value region. This suggests that the strain gauge in this particular is located in the area dominated by lower strain values, randomly selected within the measurement area. In the case of the second test (Figure 10), the curve runs more towards the middle, indicating that the measurement is more averaged.

There is a noticeable peculiarity in the strain curves (Epsilon X), clearly visible in Figure 10 at approximately the 7-s mark. At that specific moment, a significantly increased localised strain began to emerge in the analysed faced fields, indicating the occurrence of a microcrack within the sample. This crack is visibly depicted as a red area within the selected facet fields, as illustrated in Figure 8. It is worth noting that this red area is absent at the beginning of the test and starts to manifest around the 7 s mark. Additionally, the crack specifically manifests in the X direction and does not appear in the Epsilon Y curves. Furthermore, the strain gauge measurement does not capture this crack as the T-rosette is positioned in a different area of the sample.

The relative differences between the high-value and average-value areas, as well as between the low-value and average-value facets, were analysed. Table 3 presents the results for the test at a loading rate of 0.02 mm/min, with values determined at 5000, 10,000, and 15,000 s. Table 4 presents analogous results for 5, 10, and 15 s of testing at a loading rate of 20 mm/min.

Differences in strain measurements depending on the selected facet groups can reach several percent. At the beginning of the test, with a loading rate of 0.02 mm/min, choosing facets from a high-value area resulted in approximately 6% higher strains in the X direction compared to a balanced selection (average-value area). On the other hand, selecting extremely low-strain fields led to measurements underestimating the values by over 12% compared to the averaged values. As the test progressed, the relative differences increased. After 10,000 s, a relative difference for the high-strain field reached nearly 14%, while the low-strain field underestimated the values by 16%. It is important to note that the difference between the highest and lowest strain fields also increased. During the subsequent reading at 15,000 s, the differences stabilised and slightly decreased.

The analysis of transverse strains confirms the aforementioned trend. The differences in the second reading reach up to 20% for the lowest strain field. However, it is worth noting that the differences are lower compared to the axial direction.

**Figure 11:**
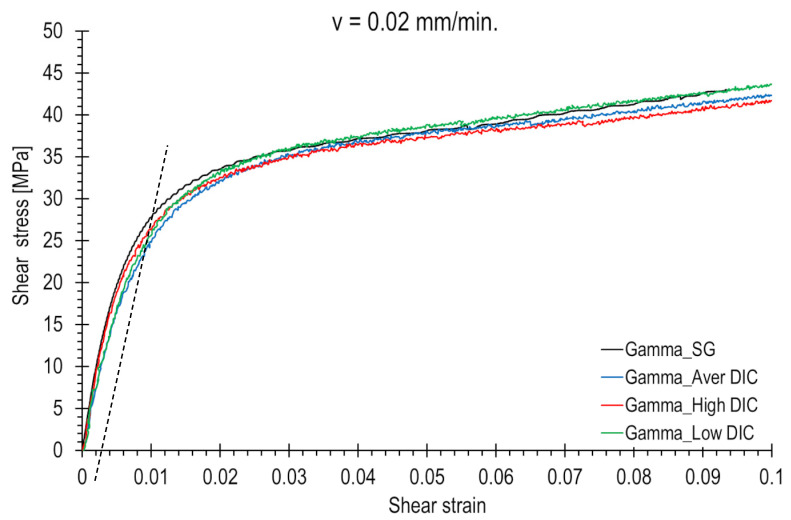
Shear stress–shear strain curve waveforms for different strain measuring methods and various facet areas. The loading rate is equal to 0.02 mm/min. The shear offset yield strength was determined using dashed lines.

**Figure 12:**
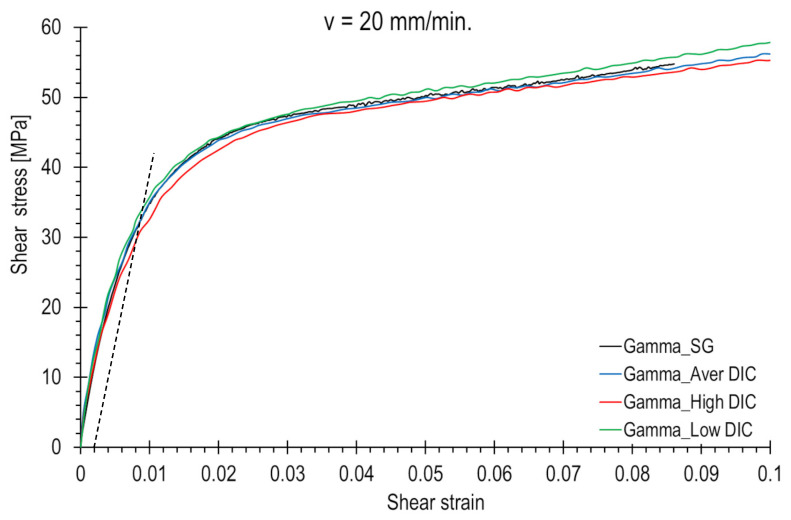
Shear stress–shear strain curve waveforms for different strain measuring methods and various facet areas. The loading rate is equal to 20 mm/min. The shear offset yield strength was determined using dashed lines.

The results obtained from the strain gauge measurement method and various DIC facet areas were used to generate shear stress–strain curves, which are presented in Figure 11 and Figure 12. In all cases, the shear strain values were calculated using Equation (1). These graphs illustrate how the location of the strain measurement area influences the shape of the shear stress curve. The electro-resistant curve (black colour) generally lies roughly between the high- and low-value DIC curves.

Noteworthy differences can be observed, particularly in the transitional region where the shear curve shifts from linear to nonlinear. This can have a significant impact on the measurement of strength parameters, such as the shear offset yield strength. For a loading rate of 20 mm/min, readings can range from 27 to 32 MPa (indicated by the dotted line in Figure 12), depending on the selection of the measurement area or the positioning of the strain gauge. At a lower loading rate, the values can fluctuate between 21 and 27 MPa (indicated by the dotted line in Figure 11).

It can be concluded that choosing a location on the sample surface with a lower level of strain results in an increase in the shear stress curve because the given load value is recorded for a lower strain. The electro-resistant curves lie roughly between the upper and lower DIC curves. In the DIC analysis, it is possible to choose any area. The choice of the location for strain gauge mounting is random. 

## 4. Discussion of the Results

The determined material properties of the G/V composite, as a function of strain rate, indicate that the shear strength increases with higher strain rates. This relationship can be represented by a straight line on logarithmic loading rate coordinates. Such a trend is commonly observed in polymer matrix composites that demonstrate viscoelastic behaviour and is consistent with findings reported in the existing literature.

The investigation of the shear stress–strain curve was extended with optical measurements, enabling the determination of strain distribution on the G/V composite. An uneven distribution of the strain field was observed, characterised by a net-like pattern caused by the presence of surface layers of glass fabric in the composite.

Based on measurements from various facet areas, it was observed that there are relative differences of several percent between the areas of the averaged facets and the areas of the facets with high or low values. These differences are more pronounced at higher loading rates, where they can reach up to 25%.

DIC measurement is well suited for detecting anomalies and damaged areas in FRP composites, as investigated in [32]. During the G/V test, it was observed on the DIC maps that as the damage progresses, an increasing number of areas exhibit significant levels of strain. This phenomenon can be attributed to the development of damage, formation of defects, and microcracks. This could potentially explain the non-linear shape of the shear stress–strain curve. 

**Figure 13:**
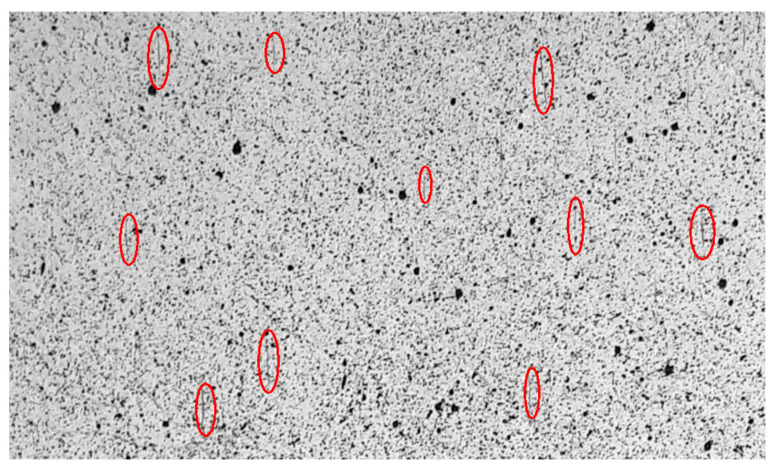
An example image of a DIC area from one of the samples tested with a loading rate of 20 mm/min reveals the presence of multiple visible cracks in the field, examples of which were indicated with the red ellipses.

In the shear graphs (Figure 11 and Figure 12), the gamma strain is limited to 10%. However, shear-by-tensile tests were carried out until failure. An example image of a DIC area from one of the samples is provided in Figure 13. Despite being observed at low magnification, numerous cracks, indicated by ellipses, are visible on the surface of the samples. Nevertheless, associating specific cracks with the corresponding DIC image can be challenging due to potential overlapping and alterations that may occur throughout a test.

Strain field optical measurement methods, such as Digital Image Correlation (DIC), appears to be the optimal choice for capturing non-uniform strain distribution on the surface of GFRP composites. When analysing DIC results, it is essential to pay attention to potential variations in the strain distribution. In the case of observing regions with higher strain values, it is important to verify whether they are a result of deformation or material damage.

The shear stress–strain curve obtained via tensile testing does not take into account the non-uniform strain distribution on the composite. Usually, the curve is determined based on strain gauge measurements. However, the installation of T-rosette strain gauges is random with respect to the described strain distribution. Consequently, the placement of the strain gauge or choosing a DIC facet area can influence the determined gamma strain and strength parameters. In particular, it may influence the measurement of the shear offset yield strength. When using the electrical resistance method, it is recommended to place multiple strain gauges on the measurement surface of the tested material in order to achieve more reliable results and understand the variability of values. This allows for the determination of strains at multiple measurement points and the identification of any areas with atypical responses.

The literature on FRP composite research is extensive, with numerous studies utilising the DIC method for strain field measurement. However, most of these studies concentrate on carbon FRP composites and cover a wide range of strength tests conducted under different conditions, making direct comparisons with this work challenging or even impossible. Nevertheless, the overall findings remain consistent. The strain field is observed to be non-uniform, as demonstrated in studies such as [26,27,33], which were dedicated to CFRP composite. During testing of the G/V, the occurrence of cracks, damage, and delamination becomes clearly visible on DIC maps.

When conducting strain measurements using both strain gauges and DIC, it is important to compare the results obtained from these methods to identify any differences and understand their causes.

## 5. Conclusions

This study aimed to investigate the non-uniformity of the strain field during the off-axis tensile test of the G/V composite and its influence on the shape of the shear curve. The following key conclusions can be drawn:

- Significant non-uniformities in strain distribution were observed on the tested samples’ surface, manifested as a mesh-like pattern.
- The choice of the measurement area had an impact on the shape of the stress–strain curve. Differences in strain measurements depending on the selected areas could reach several percent and influence the obtained stress–strain curves and strength parameters.
- The DIC method proved to be valuable in detecting anomalies and damages in FRP composites. As the damage progressed, an increasing number of regions with higher strain values exceeding two percent, were appearing on the DIC maps. Microcracks and defects were identified within the composite structure. This could potentially explain the non-linear shape of the shear stress–strain curve.
- It is essential to consider potential variations in strain distribution when analysing DIC results. In the case of observing regions with higher strain values, it is important to verify whether they result from deformation or material damage.
- When utilising the electrical resistance method, it is recommended to place multiple strain gauges on the measurement surface of the tested material in order to achieve more reliable results and understand the variability of values. This allows for the determination of strains at multiple measurement points and the identification of any areas with unusual responses.
- When conducting strain measurements using both strain gauges and DIC, it is important to compare the results obtained from these methods to identify any differences and understand their causes.

## Figures and Tables

**Table 1:** Mean values and standard deviations for in-plane shear modulus G_12_ and in-plane shear strength R_12_ of the G/V composite [4,34].

Parameter, Standard	Unit	Value
Viscosity at 25 °C, DIN 53015	[mPa · s]	300 ÷ 500
Gelation time at 25 °C, ISO 2535	[min]	10 ÷ 30
Tensile strength, ISO 527-2	[MPa]	80
Relative elongation at break, ISO 527-2	[%]	3.5
Tensile modulus, ISO 527-2	[MPa]	3500
Heat distortion temperature under load (HDT), ISO 75-2	[°C]	85

**Table 2:** Mean values and standard deviations for in-plane shear modulus G_12_ and in-plane shear strength R_12_ of the G/V composite [4].

Loading Rate	In-Plane Shear Modulus G_12_	Standard Deviations G_12_	In-Plane Shear Strength R_12_	Standard Deviations R_12_
[mm/min]	[mPa]	[mPa]	[mPa]	[mPa]
0.02	3395	74	38.4	0.6
2.0	3885	109	47.4	1.9
20.0	3952	194	49.3	1.6
2000	3502	100	58.2	3.1

**Table 3:** Analysis of relative differences in directional strain measurements with different measurement fields selected. The loading rate is set at 0.02 mm/min.

Time	Epsilon_X_aver_	Epsilon_X_max_	Epsilon_X_min_	The Difference Relative to X_aver_ of	
[s]	[mm/mm]	[mm/mm]	[mm/mm]	X_max_	X_min_
5000	0.0163	0.0173	0.0143	6.2	−12.4
10,000	0.0338	0.0385	0.0283	13.9	−16.2
15,000	0.0502	0.0553	0.0430	10.3	−14.3
**Time**	**Epsilon_Y_aver_**	**Epsilon_Y_max_**	**Epsilon_Y_min_**	**The Difference Relative to Y_aver_ of**	
**[s]**	**[mm/mm]**	**[mm/mm]**	**[mm/mm]**	**Y_max_**	**Y_min_**
5000	−0.0133	−0.0136	−0.0118	1.9	−12.9
10,000	−0.0304	−0.0333	−0.0261	9.6	−21.6
15,000	−0.0472	−0.0494	−0.0419	4.5	−15.1

**Table 4:** Analysis of relative differences in directional strain measurements with different measurement fields selected. The loading rate is set at 20 mm/min.

Time	Epsilon_X_aver_	Epsilon_X_max_	Epsilon_X_min_	The Difference Relative to X_aver_	
[s]	[mm/mm]	[mm/mm]	[mm/mm]	X_max_	X_min_
5	0.0141	0.0158	0.0106	12.0	−25.0
10	0.0311	0.0360	0.0232	15.9	−25.4
15	0.0485	0.0553	0.0376	14.1	−22.6
**Time**	**Epsilon_Y_aver_**	**Epsilon_Y_max_**	**Epsilon_Y_min_**	**The Difference Relative to Y_aver_**	
**[s]**	**[mm/mm]**	**[mm/mm]**	**[mm/mm]**	**Y_max_**	**Y_min_**
5	−0.0093	−0.0101	−0.0112	8.3	11.6
10	−0.0256	−0.0232	−0.0247	−9.4	6.4
15	−0.0402	−0.0388	−0.0406	−3.5	4.6

## Data Availability

The necessary data are contained within the article.
